# The Evolutionary History of a DNA Methylase Reveals Frequent Horizontal Transfer and Within-Gene Recombination

**DOI:** 10.3390/genes14020288

**Published:** 2023-01-21

**Authors:** Sophia P. Gosselin, Danielle R. Arsenault, Catherine A. Jennings, Johann Peter Gogarten

**Affiliations:** 1 Department of Molecular and Cell Biology, University of Connecticut, Storrs, CT 06268-3125, USA; 2 Institute for Systems Genomics, University of Connecticut, Storrs, CT 06268-3125, USA

**Keywords:** actinophage, actinobacteriophage, inteins, LAGLIDADG endonuclease, homing, horizontal gene transfer, DNA methyltransferase, homologous recombination, selfish genetic elements

## Abstract

Inteins, often referred to as protein introns, are highly mobile genetic elements that invade conserved genes throughout the tree of life. Inteins have been found to invade a wide variety of key genes within actinophages. While in the process of conducting a survey of these inteins in actinophages, we discovered that one protein family of methylases contained a putative intein, and two other unique insertion elements. These methylases are known to occur commonly in phages as orphan methylases (possibly as a form of resistance to restriction–modification systems). We found that the methylase family is not conserved within phage clusters and has a disparate distribution across divergent phage groups. We determined that two of the three insertion elements have a patchy distribution within the methylase protein family. Additionally, we found that the third insertion element is likely a second homing endonuclease, and that all three elements (the intein, the homing endonuclease, and what we refer to as the ShiLan domain) have different insertion sites that are conserved in the methylase gene family. Furthermore, we find strong evidence that both the intein and ShiLan domain are partaking in long-distance horizontal gene transfer events between divergent methylases in disparate phage hosts within the already dispersed methylase distribution. The reticulate evolutionary history of methylases and their insertion elements reveals high rates of gene transfer and within-gene recombination in actinophages.

## 1. Introduction

The SEA–PHAGES (Science Education Alliance–Phage Hunters Advancing Genomics and Evolutionary Science) program organizes undergraduate courses in which students isolate, sequence, and annotate genomes of phages that infect actinobacteria. The SEA–PHAGES program is organized by Graham Hatfull’s group at the University of Pittsburgh and the Howard Hughes Medical Institute’s Science Education division [1]. As part of the bioinformatics section of the SEA–PHAGES course at the University of Connecticut, we developed a course-based undergraduate research experience in which students characterized inteins and their evolution in actinophages. 

**Inteins**, aka protein introns, are selfish genetic elements similar to self-splicing introns; the difference is that they are transcribed and translated together with the host protein (extein), and only remove themselves following translation [2,3,4,5,6]. Typical inteins have two domains: the self-splicing domain acts to rejoin the two parts of the host protein, and the homing endonuclease domain allows for the intein to invade previously uninvaded alleles. The homing endonuclease has a site specificity that corresponds to the intein insertion site and the surrounding nucleotides. It makes a double strand cut in the uninvaded allele at the site where the intein coding sequence is to be inserted. The host’s machinery then repairs the double strand break using the invaded allele as a template. Importantly, inteins do not have their own mechanism to jump from one organism to another; rather, they rely on the flow of genetic information that occurs by other mechanisms. When two homologous genes are present in a single cell with one being an intein-free copy and the other being an intein-containing copy, the translated intein facilitates the invasion of the intein-free allele with high efficiency [7,8]. Inteins invading the same site are known as intein alleles. Intein alleles from different organisms that invade the same site in a gene are much more similar to one another than to inteins invading the same organism but at different sites or genes. Typically, inteins invade in conserved regions of conserved protein-coding genes [9]. This is also true for inteins in actinobacteriophages [10].

**Actinobacteriophages**, or actinophages, are viruses that infect actinobacteria [11], a bacterial phylum that includes many soil bacteria (e.g., species in the genera *Streptomyces* and *Microbacterium*), but also important pathogens (e.g., *Mycobacterium tuberculosis* and *M. abscessus*). One driving force behind the study of actinophages is their potential use in phage therapy [12,13]. The discovery of phages that can lyse bacterial cultures led d’Herelle, one of the co-discoverers of bacteriophages, to use them to combat bacterial infections [14]. The ability of phages to effectively attack Gram-positive bacteria is one of the motivations for the SEA–PHAGES project. On 12 December 2022, the PhagesDB databank [15,16] reported on 22,387 actinobacteriophages, of which 4184 had complete genome sequence records, which were in turn divided into 148 clusters of related phages and 62 singletons, i.e., phages that currently are not members of a cluster. The proteins encoded in these genomes are grouped into families, called phams or phamilies, based on sequence similarity [17]. In general, current and past research finds that these phamilies exhibit typical patterns of intein invasion [9]. Inteins are found in important genes such as helicases, terminases, and many other proteins essential for capsid structure, DNA replication, and packaging [10].

One exception to the preference for conserved and important proteins that we found was an intein in a gene annotated as a putative DNA methylase. In addition to the intein, a few members of the analyzed methylase family contain another region encoding a nearly identical protein sequence fragment. We refer to this fragment as the ShiLan domain, as the sequence was first discovered in the ShiLan phage. The ShiLan domain is present in otherwise divergent members of the gene family. A third sequence present in only a few members of the methylase family encoded an additional homing endonuclease of the LAGLIDADG family. This endonuclease was different from the LAGLIDADG homing endonuclease associated with the intein and exhibited significant similarity to a homing endonuclease in a group I intron.

**DNA methylases** play diverse roles. They often are part of restriction modification systems (RMSs), play a role in marking DNA regions, and are critical for mismatch repair and regulation of the origin of replication in bacteria [18,19]. RMSs are often considered part of a bacterial defense system, recognizing and restricting DNA with a different methylation pattern. However, RMSs are also a form of addiction cassette, encoding a toxin–anti-toxin system [20,21]. In this case, the restriction enzyme is the toxin and the methylase is the antitoxin. If the associated methylase is lost from a cell’s RMS, the remaining restriction enzyme activity will destroy the organism’s genome. For an RMS to be lost, first the restriction activity needs to decay; only then can the whole system be deleted. In line with their characterization as addiction cassettes, RMSs are frequently encoded on plasmids and often have a disjunct distribution (e.g., [22,23]). RMSs come in four different varieties [24]. Type I RMSs are composed of three different polypeptides acting as a single complex. One peptide acts as a specificity recognition protein, and the other two act to modify or cleave the bound DNA. Type II RMSs are the simplest true RMSs, consisting of two separate proteins (one endonuclease and one methyltransferase) that can act independently of each other and of a specificity protein. Type III RMSs form a complex such as Type I, but lack the associated specificity protein. Lastly, there are Type IV RMSs which lack a modification protein entirely, and therefore do not count as true RMSs. However, there are some exceptions to this schema, where multiple activities are encoded on a single peptide (e.g., Type IIB RMSs [25]).

Our analysis of methylase sequences reveals a sporadic distribution of the methylases, frequent transfer of genes between phages belonging to different clusters, a surprising number of recombination events between the methylase sequences from divergent phages, and a recent intein invasion of phages isolated from the same geographical area. 

## 2. Materials and Methods

### 2.1. Intein Discovery and Dataset Construction

Sequence and metadata were retrieved from PhagesDB [15]. This database contains genomes from over 4000 actinobacteriophages. PhagesDB places phages into clusters and subclusters based on their genome content (specifically, gene content dissimilarity [26]). Protein-coding genes are placed into phams, also referred to as phamilies, based on sequence identity and BLASTP search results. These phams serve as the basis for the clustering process mentioned above and are useful to retrieve homologous sequences; however, the assignment of sequences to phams and the numbering of phams changes as PhagesDB expands. For annotated phages, i.e., no longer in draft status, the protein-coding genes are uniquely identified by the name of the phage, followed by the number of the open reading frame. This identification scheme is used throughout this manuscript and allows for the retrieval of the individual genes from PhagesDB. Genomes sequenced by the SEA–PHAGES program are also submitted to the NCBI. Individual genes can be retrieved using the phage-name_number of the gene as query at https://www.ncbi.nlm.nih.gov/gene/ (accessed 10 January 2023). For example, Dorothy_75 or PopTart_63 as query retrieves the genes linked to YP_009592050.1 and YP_009214423.1, respectively. Each gene in PhagesDB is linked to its pham, and all homologs assigned to the pham can be readily downloaded; however, as the pham numbers change over time, the phage name and ORF number provide a stable way to find the gene and associated pham in PhagesDB.

The initial discovery of the insertion containing methylase sequences in phages Dorothy and Cactusjack occurred during visual inspection of viral genomes via Phamerator [27]. This initial finding was followed by repeated searches for inteins in the PhagesDB database using BLAST and psi-BLAST [28]. Intein harboring methylases from these searches belonged to phams 106461, and 105558 (as of 18 May 2022). The sequences for these phams are available in the Supplementary Materials (Supplementary Material files Pham_105558(05_18_2022).txt and Pham_106461(05_18_2022).txt). These phams were used as our source of sequence data going forward. All sequences used in this research were downloaded from PhagesDB on 18 May 2022; metadata on the phages were updated on 31 August 2022. In-house scripts used to construct local databases and extract metadata can be found at https://github.com/sophiagosselin/Methylase_Insertions (accessed 10 January 2023). HHPred [29] was used to ascertain the potential identities of the various insertion elements. Analyses were performed using the default settings of the webserver at https://toolkit.tuebingen.mpg.de (accessed on 8 December 2022). Searched databases were PDB_mmCIF70_12_Aug, Pfam-A_v35, NCBI_conserved_Domains(CD)_v3.19, and TIGRFAMs_v15.0.

### 2.2. Sequence Alignments

To perform downstream analyses, we first aligned the methylase sequences using MAFFT (v7.471) [30]. MAFFT was used to create two different alignments. The first (which we refer to as the compact alignment) used the globalpair and reorder settings, and a maximum iteration count of 1000, while the second (which we refer to as the gappy alignment) used the globalpair and reorder settings, a maximum iteration count of 1000, and an unalignlevel of 0.8. SeaView (v5.0.4) [31] was used to inspect alignments and to then define four separate site sets: one for the methylase excluding the insertion elements and one each for the three insertion elements. We will refer to the site set containing only the methylases and not the insertion elements as the methylase extein. The methylase extein set was copied and split into three different subsets. Each one contained only the methylase sequences which were invaded by a given insertion element such that there was a subset for intein-containing methylases, a subset for ShiLan domain-containing methylases, and a subset for endonuclease-containing methylases. The alignment of these three extein sub-datasets was the same as in the compact alignment.

### 2.3. Phylogenetic Tree Construction and Divergence Comparison

These alignments were then used to construct phylogenetic trees using IQ-TREE (v2.1.3) [32]. As the different components of the methylase sequences (extein, intein, ShiLan domain, and second homing endonuclease domain) likely had different evolutionary histories, we estimated the best fitting model for each alignment separately using IQ-TREE’s built-in ModelFinder. Table 1 lists the models selected for each dataset. Bootstrap support was created for each tree using the ultrafast bootstrapping option with 1000 bootstraps. Resulting phylogenies were visualized in Figtree (v1.4.4) and then editorialized in vector graphics software (InkScape (v2.2)). The maximum likelihood tree for the methylase extein sequences was also used as in the AU-test (below).

To compare sequence divergence rates between the methylase and the various insertion elements, phylogenies were constructed for each of the three insertion elements and for the three extein sub-datasets listed above. Each of these six trees were constructed in the same manner previously described. These trees were then compared by computing the pairwise maximum likelihood distances between the tips of the tree using IQ-TREE. The corresponding matrices (i.e., intein containing methylases only, and the inteins only phylogenies) were compared by calculating the correlation between these pairwise distance matrices via Microsoft Excel (v16.67).

### 2.4. Approximately Unbiased (AU) Test

Constraints for the AU-test, i.e., unresolved trees that represented the constraints, were created in a text editor, and the best maximum likelihood (ml) tree given these constraints was then constructed in IQ-TREE [32] using the–g option and constrained Newick trees. Constrained maximum likelihood trees were built such that the clan of interest was constrained to only contain members of this group, but all other taxa could be freely placed. 

### 2.5. Protein Structure Prediction

A predicted protein structure was generated for the PopTart_63 methylase, which contains no insertion elements. This methylase sequence was used as input for the AlphaFold v2.2.4. [33] Jupyter notebook hosted on Google Colab. The predicted structure was then colored in Chimera [34] to indicate the insertion sites of the ShiLan domain, intein, and second homing endonuclease. In addition, AlphaFold v2.2.4 was used to generate a predicted structure for the full methylase from the Taj phage. The Taj methylase does not contain the ShiLan domain nor the intein, but does contain the second homing endonuclease. The predicted structure was colored in Chimera to indicate the three insertion sites and the second homing endonuclease domain.

## 3. Results

We analyze the evolutionary history of intein-containing methylases in actinobacteriophages, including both intein-containing and intein-free homologs. In addition to the intein, we find and analyze two additional insertion sequences with sparse distribution in the studied methylase family: a second homing endonuclease not associated with the intein, and a unique domain we refer to as the ShiLan domain. We use 271 of these methylases spanning thirteen phage clusters and three singleton phages to reconstruct the evolutionary histories of both the methylases and their insertion elements to identify gene transfer and recombination events. We also compare the divergence between the insertion elements to the divergence between the methylases to identify recent local invasion events.

### 3.1. Distribution of Methylases Similar to Dorothy_75

**Figure 1:**
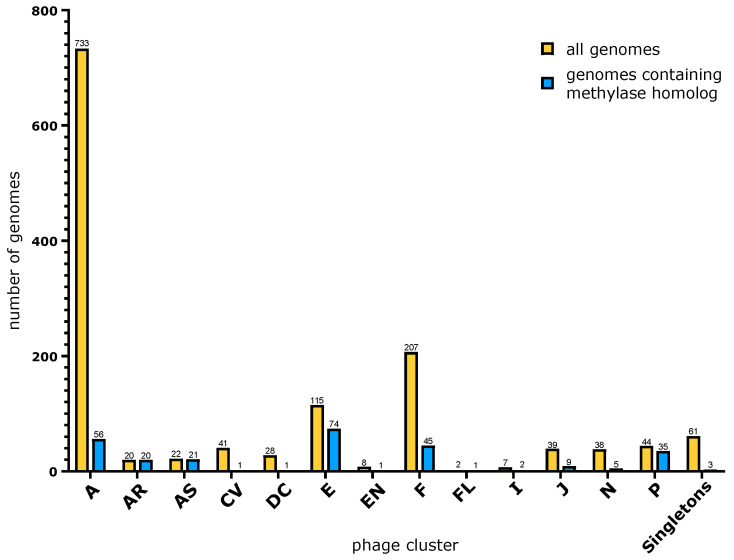
Bar graph depicting the total number of genomes per phage cluster in PhagesDB as of 27 December 2022 (**yellow**) and the number of genomes encoding a methylase in our dataset (**blue**). The clustering of phages in PhagesDB reflects overall nucleotide similarity, gene order, and gene content [26]. See the Methods Section for details.

The first intein containing methylase we identified was Dorothy_75. Homologous intein-harboring methylases were identified in phams 106461 and 105558 (18 May 2022). Pham 106461 has 17 members. This pham contained most of the intein-containing homologs; however, some intein-containing methylases had been placed into pham 105558, with 254 members. These two phams undoubtedly contain homologous sequences. A pairwise comparison in PRSS [35] between the methylase from ShiLan (ShiLan_65, placed in the second cluster) and Dorothy_75 resulted in a 938 aa overlap with a Z-score of 3833 and an E(10,000) value of 7 × 10–169. The E(10,000) value gives the expectation of the number of matches with the same or better quality if 10,000 shuffled sequences are compared. For the following analyses, the two phams were combined. These homologous methylases have a wide distribution among actinophages. They are found in thirteen different phage clusters and in three singletons (Figure 1). However, these methylases have a sparse distribution. In only one of the clusters is the methylase present in all members of the cluster, and in most clusters the phages without the methylase gene outnumber the ones that encode the methylase in their genome.

In the genome of phage Dorothy, the open reading frame (ORF) next to Dorothy_75, Dorothy_76, is also annotated as methylase. This ORF has homologs in 185 other genomes, e.g., Taj_80 and Poptart_64. These ORFs are short (44 amino acids in Dorothy_76), and the rational for the annotation as methylase is not evident. Most of the homologs detected through blastp searches at PhagesDB and NCBI are annotated as hypothetical proteins (NCBI) or “function unknown” (PhagesDB). In HHpred using Dorothy_76 as query, no matches with E-values smaller than 1.7 were recovered. This small ORF has a wider distribution (65 homologs in phages of the F-cluster), compared to the 46 methylases that are the focus of this study.

Many actinobacteriophages encode more than one methylase. For example, in phage PopTart ORF 60 and 63 are annotated as methylases. PhagesDB placed these two methylases into different phams. PRSS detects two regions of similarity between the two methylases with E(10,000) values of 7.6×10^−6^ and 0.24. The following analyses do not include these more divergent methylases.

### 3.2. Alignments of Phage Encoded Methylase Genes and Their Insertion Sequences

For our analyses we used two different alignments. One is a more compact traditional multiple sequence alignment (MSA), and the other is an alignment that aligns uncertain alignment regions to gaps in the other sequences. In the following, we label these as the compact and gappy alignments, respectively. The gappy alignment focuses on reliably aligned residues, minimizes potential artifacts from the guide tree, and results in phylogenies with much shorter branch lengths since gaps in the other sequences are encoded as missing data. 

**Figure 2:**
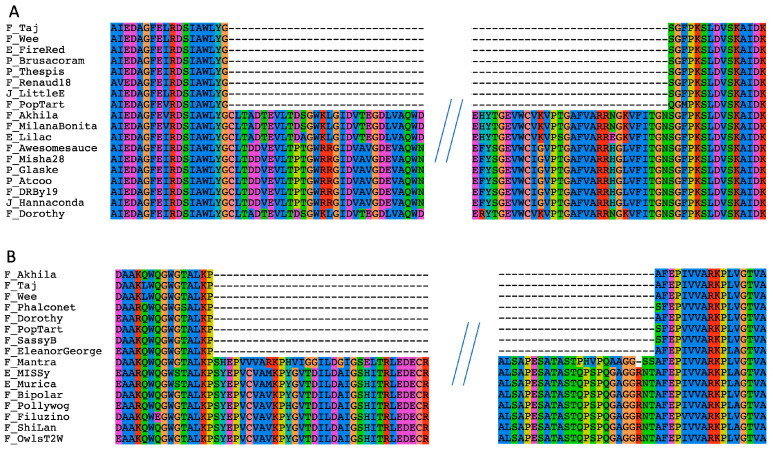
Alignment of the beginning and end of the intein (panel **A**) and the ShiLan domain (panel **B**) with their associated surrounding regions. In the Dorothy phage, the intein is 327 amino acids long. In the ShiLan phage, the ShiLan domain is 202 amino acids long.

**Figure 3:**
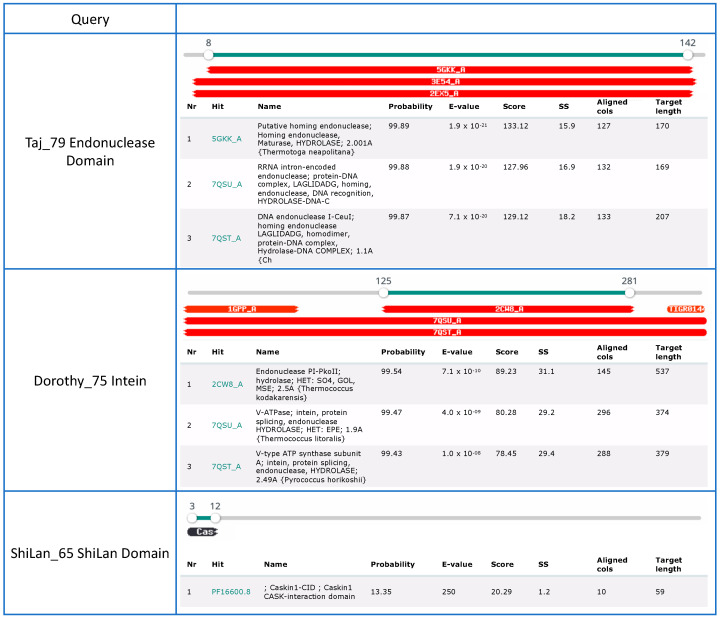
Results from HHPred searches using the three inserted sequences as queries. Only the three most probable matches are given. The endonuclease domain exhibits significant similarity to endonucleases of the LAGLIDADG type. The intein over its whole length matches intein sequences (match two and three). The search with the ShiLan domain did not result in any significant matches.

Some of the methylases had been identified as intein-containing using a PSI BLAST search. The multiple sequence alignment of the methylase sequences revealed that 21 of them contain an intein in the same position. The following findings confirm the identity of this insertion as an intein:

- The intein is present in only a fraction of the sequences;
- It is inserted in a conserved region of the methylases (Figure 2A);
- Results from an HHPred search show homology to inteins over the whole length of the insertion (Figure 3);
- The intein sequences have a phylogeny different from the remainder of the methylase (see below).

In addition, we found another insertion present in ShiLan_65 and seven other methylases. Going forward we will refer to this insertion as the ShiLan domain. The sequence of this insertion is conserved, is inserted in a conserved region of the methylase (Figure 2B), and is found in divergent methylases of phages from the E and F clusters. An HHPred search using this sequence as a query resulted in only one low quality match to the beginning of the insertion sequence (Figure 3). 

In an unrelated project, one of us (DRA) conducted a BLAST search against the NCBI Virus database using the LAGLIDADG homing endonuclease contained in a group I intron (accession # YP_005089794) as query. This intron is located in in the *atpA* gene of a *Dunaliella salina* chloroplast genome (accession # NC_016732). This homing endonuclease had a significant hit to the Taj_79 methylase. Inspection of the match and the MSA of the methylases revealed that this endonuclease domain was well conserved in nine methylase sequences. Each of these methylases contained the typical motif of a LAGLIDADG endonuclease; however, this endonuclease domain was located outside and upstream of the intein insertion site. In an HHPred search, this endonuclease had significant matches to homing endonucleases (Figure 3).

**Figure 4:**
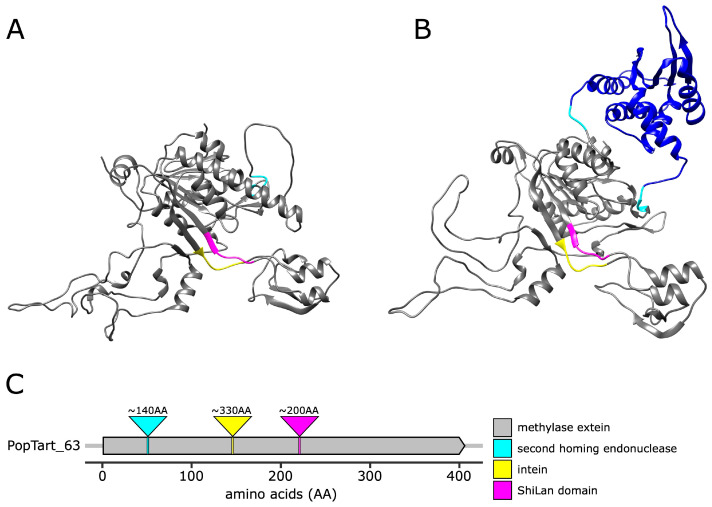
Location of insertion sites of the second LAGLIDADG homing endonuclease domain, the intein, and the ShiLan domain mapped onto predicted methylase structures (panels **A** and **B**) and the linear sequence of insertion-free methylase PopTart_63 (panel **C**). The structure of the PopTart_63 (panel **A**) and Taj_79 (panel **B**) methylases were predicted by AlphaFold v2.2.4. Three residues upstream and downstream of each element’s insertion site are indicated as follows: ShiLan domain in magenta, intein in yellow, and the second homing endonuclease in cyan. The putative homing endonuclease domain in Taj_79 is in blue. The model confidence for the two structures is depicted in Figure S2. Panel C gives the approximate size and location of the insertions mapped onto the PopTart_63 gene. The second homing endonuclease domain and the intein in Akhila_67 have a length of 139 and 327 amino acids, respectively. The ShiLan domain in Mantra_64 is 202 amino acids long.

The locations of each element’s insertion site were visualized using AlphaFold-predicted structures of the PopTart_63 methylase, which does not contain any of the three insertion elements, and Taj_79, which contains the endonuclease insertion (Figure 4).

### 3.3. Methylase Phylogeny

#### 3.3.1. Methylases Do Not Group According to the Cluster to Which the Phages Belong

**Figure 5:**
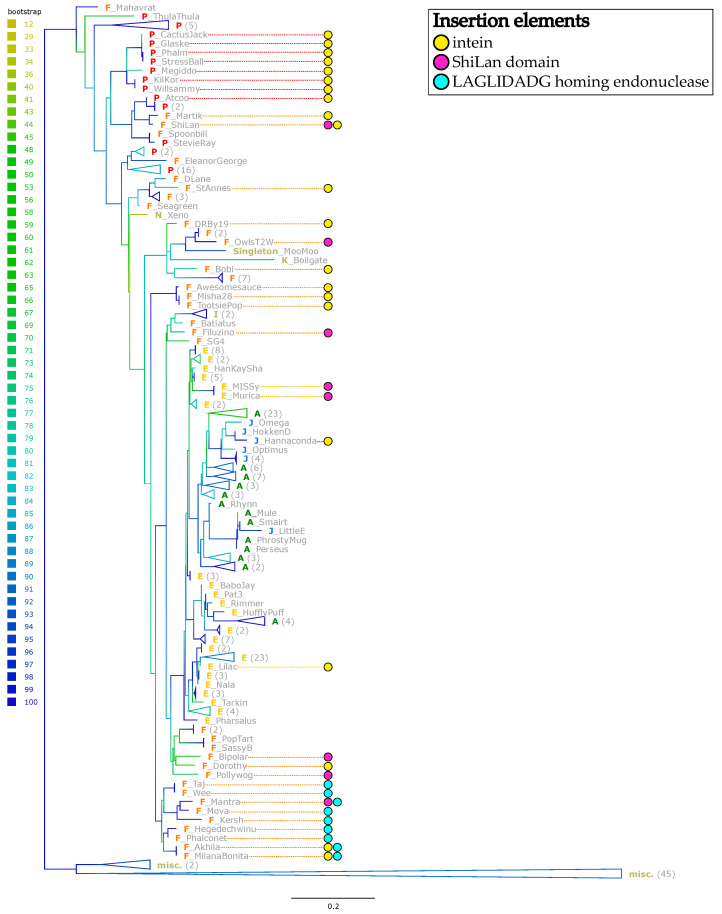
Phylogeny of the methylase family without considering the insertion sequences. The phylogeny was calculated with IQ-TREE from the gappy alignment with the intein, ShiLan, and second endonuclease domain removed. The cluster to which each phage belongs is denoted by a colored prefix in bold. Sequences that contain the intein (**yellow**), the ShiLan domain (**magenta**) and the second homing endonuclease domain (**cyan**) are indicated by colored circles. Branches are colored to reflect bootstrap support values. Neighboring insertion-free sequences belonging to the same phage cluster were collapsed where possible to increase readability. Numbers in parentheses indicate the number of taxa contained within the collapsed group. Collapsed groups labeled “misc.” contain insertion-free taxa from several phage clusters (AR, AS, CV, DC, EN) and three singletons. Supplementary Figure S1B contains the phylogeny without collapsed branches.

The maximum likelihood phylogenies (Figure 5 and Supplementary Figure S1A,B) reconstructed from the compact and gappy alignments are similar in that:

- The sequences from phage clusters DC, CV, FL, AR, and AS together with two sequences from separate singletons form a well-supported clan in both phylogenies (a clan is a group of tips that group together in an unrooted phylogeny, corresponding to a clade in a rooted phylogeny);
- The sequences from clusters F, P, E, A, and J do not form clans.

The two phylogenies differ in that:

- Details of the branching order are not consistent between the two topologies;
- The two phylogenies have different estimated branch lengths.

We used KH [36], SH [37], and AU [38]-tests, as implemented in IQ-TREE2 [39] to determine whether the possibility of the sequences from each cluster grouping together could be rejected with confidence. Bias created through the alignment process tends to reinforce the clusters from the guide tree. To minimize the effect of alignment bias, we chose the gappy alignment for this analysis. The maximum likelihood phylogeny constrained to group all the clusters as individual clans was rejected, as was the maximum likelihood phylogeny that only constrained methylase from cluster F as a clan. The results are summarized in Table 2.

#### 3.3.2. Intein and ShiLan Domain-Containing Methylases Do Not Form Clans

Figure 5 depicts the maximum likelihood phylogeny calculated from the gappy alignment of the methylase family. The sequences containing the intein, the ShiLan domain, and the second endonuclease domains are indicated. The intein and ShiLan domain-containing sequences do not cluster together, whereas the sequences with the second endonuclease domain are restricted to the F-cluster and group together as a clan.

We calculated the best maximum likelihood trees which constrained each of the three types of insertion sequences to its own clan. The trees constraining the intein or ShiLan domain-containing sequences into a clan were confidently rejected as being part of the confidence set (Table 2). In contrast, the phylogeny constraining the sequences into a clan that harbors the second homing endonuclease domain was not rejected.

#### 3.3.3. Comparison of Phylogenies for the Inserted Elements and the Methylases That Harbor These Elements

**Figure 6:**
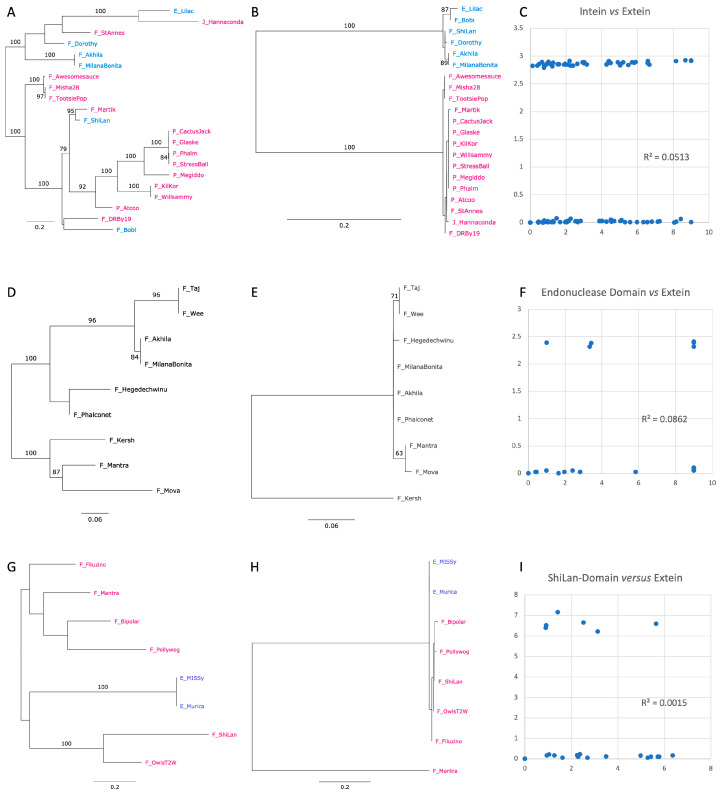
Phylogeny of subsets of the methylase family (panels **A**,**D**,**G**) compared to the phylogenies of the intein (**B**), second endonuclease, (**E**) and ShiLan domain (panel **H**). The phage names in panels (**A**,**B**) are colored to reflect the two intein groups. Phage names in panel (**G**,**H**) are colored to reflect the clusters to which the phage belongs. Panels (**C**,**F**,**I**) plot the pairwise maximum likelihood (ml) distances for the inserted element against the ml distances of the extein sequence.

Phylogenies for the intein, ShiLan, and second endonuclease domains were compared to the extein sequences (minus the intein, ShiLan, and second endonuclease domains) that harbored the respective elements (Figure 6). To better capture the divergence of the sequences, the constrained alignment was used for these comparisons. The intein sequences fall into two well-supported groups (colored blue and red in Figure 6B); these two groups do not form clans in the extein phylogeny (Figure 6A). The divergence within the two intein groups is also much lesser than the divergence between the extein sequences (in Figure 6A the names are colored according to the two intein groups).

For both the second endonuclease (Figure 6E) and the ShiLan domain (Figure 6H), one of the sequences (from phages Kersh and Mantra, respectively) is more divergent, while the remaining sequences are much more similar to one another. These more related sequences are less divergent from one another than the methylase sequences in which they are found. For all three elements, the distances between the elements do not correlate with the distances between the methylases that contain said elements (Figure 6C,F,I). The R squared values are 0.051, 0.086, and 0.001 for the correlation between intein and extein, endonuclease and methylase, and ShiLan domain and methylase, respectively. However, when the distances to the most divergent endonuclease domain (from phage Kersh) are excluded, the R squared for the second endonuclease increases to 0.69, whereas the R squared for the ShiLan domain with the distances to phage Mantra omitted remains low at 0.12.

Phages CactusJack, Glaske, Phalm, StressBall, Megiddo, KilKor, and Willsammy were isolated in Texas at LeTourneau University; sequences from these phages group together in both the extein and intein phylogenies (Figure 6A,B). The inteins in these phages are identical (Figure 6B), whereas the extein sequences exhibit divergence (Figure 6A). Similarly, phages TootsiePop and Misha28 were isolated from Massachusetts, and Awesomesauce from Rhode Island. These phages group together in both the intein and extein phylogenies; however, the inteins are identical, whereas the exteins exhibit minor sequence divergence.

## 4. Discussion

The family of putative DNA methylases on which we report here has a sporadic distribution in the clusters of actinophages (Figure 1). These methylases are surprisingly divergent for a gene invaded by an intein [9]. Given this divergence, the reconstruction of the evolutionary history of these methylases must be considered with caution. The homologous methylases from phages belonging to the same cluster (clusters A, AS, AR, E, F, I, and J) do not group together (Figure 5 and Supplementary Figure S1A,B). However, statistical tests provide strong support only for the sequences from cluster F to not form a clan (Table 2). Nevertheless, the finding that the F-cluster sequences do not group together, and the fact that only a fraction of genomes in each cluster contain a homolog to this methylase, suggest that these methylases were frequently gained and lost by the phages. This observation is similar to the studies of RMSs in bacteria [22] and archaea [23], which found that RMSs are often part of the mobilome, gained though horizontal gene transfer, and not fixed in a lineage.

While some orphan methylases play important roles and are fixed in bacterial and archaeal lineages [18,19,23], many orphan methylases have a sporadic distribution similar to RMSs [22,23]. One explanation for this is that these methylases had been part of an addiction cassette/RMS [20] from which the restriction activity was deleted. This deletion of the toxin part of the system leaves the methylase activity behind, which then no longer plays an essential role and in the fullness of time will also decay and be deleted. However, in phages a more reasonable explanation is that the orphan methylases protect the phage DNA against digestion by a host’s RM system [40]. However, this strategy can be counteracted by a host’s type IV restriction endonucleases [24], which cleaves methylated DNA motifs. This arms race between a host’s type IV restriction endonuclease and the phage’s DNA methylase explains why phage methylases are among the genes most frequently found on genomic islands [41]. If a host’s RMS methylates a particular motif, this host is protected to some extent against phages that contain unmethylated versions of the motif. The acquisition of a methylase by the phage that methylates this motif will allow for the phage to also propagate in the hosts, creating a selection pressure in the host population to lose the RMS system and acquire a type IV restriction that cleaves at the methylated motif. This in turn will create a selection pressure in the phage population to lose the methylase activity. As a consequence, the phage populations are likely to vary in their complement of DNA methylases, and the bacterial host in their type IV restriction endonucleases.

The intein we investigated contains a homing endonuclease domain of the LAGLIDADG type. The second endonuclease we discovered is also a LAGLIDADG type endonuclease but is located outside the intein. It is found in a group of related phages from the F cluster (Figure 4, Figure 5 and Figure 6D). The sequence divergence for this endonuclease, with the exception of the sequence from phage Kersh, correlates reasonably well with the divergence of the extein. The significant similarity between this domain and a homing endonuclease from a group I intron suggests that this domain may represent an independent selfish genetic element that targets a region upstream of the intein insertion site; however, we do not find strong evidence for this domain to have been transferred between phage lineages (Table 2, Figure 5 and Figure 6D,E). An alternative explanation is that the endonuclease domain is part of an RMS that contains both the endonuclease and methylation activity in the same peptide [25]. If this were the case, then the presence of the second endonuclease may represent the original form of the enzyme with the second endonuclease domain being lost from most sequences. However, the latter explanation is unlikely as LAGLIDADG endonucleases are known for their long recognition sites, function in homing, and have not been described as part of RMSs. In the structure predicted for the Taj methylase (Figure 4B) the second homing endonuclease forms its own domain, and the remainder of the structure is similar to the predicted structure of the Poptart_63 methylase (Figure 4A). This suggests that the presence of this homing endonuclease domain may not interfere with the function of the methylase.

The inteins in the methylases fall into two well-supported types (blue and red labels in Figure 6B). The observation that the methylases which group together in the extein phylogeny (e.g., Phages Lilac and Hannaconda, or Martik and Shilan), harbor two different intein types reveals that the inteins jumped between divergent host proteins. The transfer of inteins between divergent phages is also illustrated by intein-containing phages Hannaconda and Lilac, whose methylases are placed in well-supported groups that otherwise do not contain inteins (Figure 5). Even in instances where several of the intein containing methylases group together and are invaded by the same type of intein, a closer inspection suggests likely recent transfer of the intein. Ignoring branch lengths, one could assume that a single intein invasion occurred at the base of the seven intein containing phages that were isolated in at LeTourneau University in Texas (CactusJack, Glaske, Phalm, StressBall, Megiddo, KilKor, and Willsammy; Figure 5). However, the methylase sequences have significantly diverged in the compact alignment by over seven substitutions per site on average (Figure 6A), whereas the intein sequences are identical. This suggests that the inteins recently spread among the phages isolated in Texas, long after their methylases had diverged.

Whereas the intein and the second endonuclease encode homing endonuclease domains that likely facilitates the invasion of alleles with an empty target site, the disjunct distribution of the ShiLan domain remains enigmatic. Nevertheless, the ShiLan domain is found in divergent methylases (Figure 5) and, similar to each of the two intein types, the ShiLan domains, with one exception, have diverged much less than the associated methylase sequences. This suggests that the ShiLan domain too was transferred between divergent methylases. This notion is also supported by the AU-test (Table 2) which strongly rejects the hypothesis that the ShiLan domain containing methylases may form a coherent group in the methylase phylogeny.

## 5. Conclusions

Recombination between viruses has long been recognized as an important process. Even before the recognition of DNA as genetic material [42], Luria had inferred recombination between phages from multiplicity reactivation [43]. Despite its prominent role in the history of molecular biology, the amount of naturally occurring recombination we find in our study may be surprising to most. The intein and ShiLan domain distributions and phylogenies reveal frequent within-gene recombination events between phages belonging to different clusters. In addition, the gene targeted for invasion has a sporadic distribution, suggesting frequent gene loss and transfer events within and between phage clusters. Furthermore, the lack of divergence of the insertion element suggests recent local invasion of related phages by the intein.

## Figures and Tables

**Table 1:** Models selected by IQ-TREE using the Bayesian information criterion (BIC) for the different datasets.

Alignment	Best Fitting Model ^$$^
Exteins ^$^ compact alignment	VT + F + R7
Exteins ^$^ gappy alignment	WAG + R5
Inteins (compact alignment)	WAG + F + I
Exteins ^$^ (intein containing, compact alignment)	Blosum62 + F + G4
ShiLan domain (compact alignment)	HIVb + F + I
Exteins (ShiLan domain containing, compact alignment)	WAG + G4
Second endonuclease domain	Q.pfam
Exteins (Second endonuclease domain containing, compact alignment)	WAG + G4

^$^ Extein sequences excluded the intein, ShiLan, and the second endonuclease domains. ^$$^ See the IQ-TREE manual [32] for detailed descriptions of the models.

**Table 2:** Results from statistical test comparing constrained maximum likelihood trees to the overall best maximum likelihood tree determined by IQ-TREE [39]. Numbers give the probability with which the tree can be considered as part of the 95% confidence set. Trees rejected as being part of the 95% confidence set are indicated in bold.

*Tree*	p-KH [36]	p-SH [37]	p-AU [38]
*ml tree*	0.319	0.84	0.372
***all clusters constrained***	**0.0006**	**0.001**	**5.41 × 10^−5^**
*cluster A constrained*	0.0947	0.325	0.0529
*cluster AS constrained*	0.46	0.928	0.612
*cluster AR constrained*	0.363	0.939	0.454
*cluster E constrained*	0.54	1	0.618
***cluster F constrained***	**0.0013**	**0.0027**	**0.00012**
*cluster E constrained*	0.23	0.609	0.171
*cluster J constrained*	0.299	0.81	0.335
*cluster I constrained*	0.418	0.921	0.484
***all intein-containing seq.***	**0**	**0**	**8.05 × 10^−54^**
***all ShiLan domain-containing seq.***	**0**	**0**	**3.11 × 10^−6^**
*all endonucl. domain-containing seq.*	0.501	1	0.549

## Data Availability

All sequence data are available at PhagesDB https://phagesdb.org (accessed on 18 January 2023).

## Supplementary Materials

The following supporting information can be downloaded at: https://www.mdpi.com/article/10.3390/genes14020288/s1, Figure S1: phylogenies calculated from the compact (A) and gappy (B) alignments. Figure S2: prediction confidence score mapped on the methylase structure. PDB and Chimera session files (can be opened in chimera (v1.16)): PopTart_63_AF_predicted_structure.py, Taj_79_AF_predicted_structure.py, PopTart_63_AF_predicted_structure.pdb, Taj_79_AF_predicted_structure.pdb, Pham_105558(05_18_2022).txt, Pham_106461(05_18_2022).txt.
