# Supplementary figures and images for: The Evolutionary History of a DNA Methylase Reveals Frequent Horizontal Transfer and Within-Gene Recombination
Source: Genes (Basel). 2023 Jan 21;14(2):288. doi: 10.3390/genes14020288 (PMC9957025; doi:10.3390/genes14020288)

label

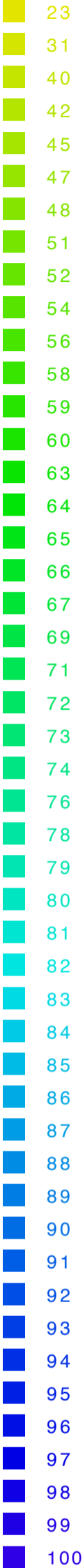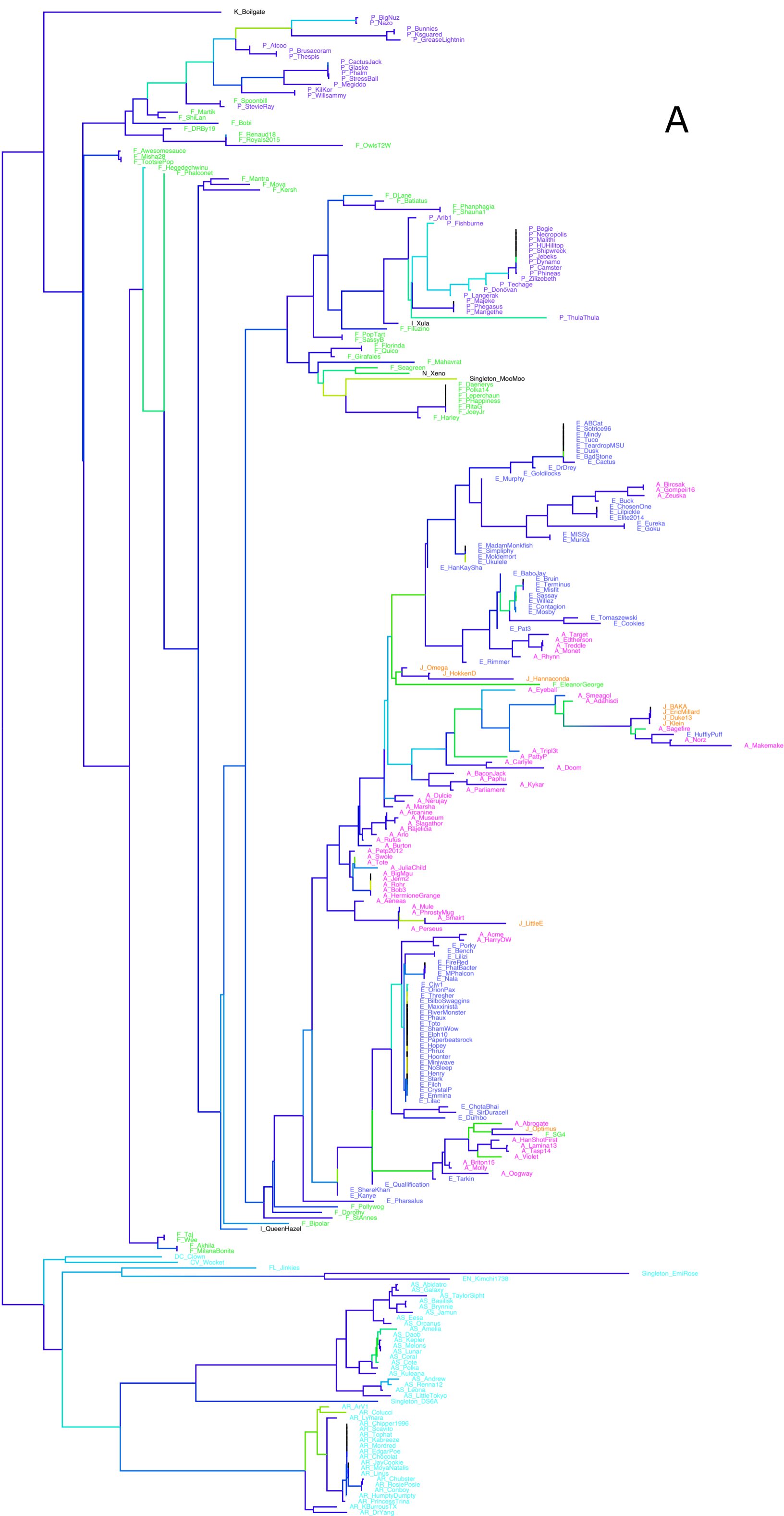

0.3

Supplement: Supplementary file 1 [file genes-14-00288-s001.zip › Figure S1A Methylase phylogeny from compact alignmnent.treefile.pdf]

label

- 12
- 29
- 33
- 34
- 36
- 40
- 41
- 43
- 44
- 45
- 48
- 49
- 50
- 53
- 56
- 58
- 59
- 60
- 61
- 62
- 63
- 65
- 66
- 67
- 69
- 70
- 71
- 73
- 74
- 75
- 76
- 77
- 78
- 79
- 80
- 81
- 82
- 83
- 84
- 85
- 86
- 87
- 88
- 89
- 90
- 91
- 92
- 93
- 94
- 95
- 96
- 97
- 98
- 99
- 100

B

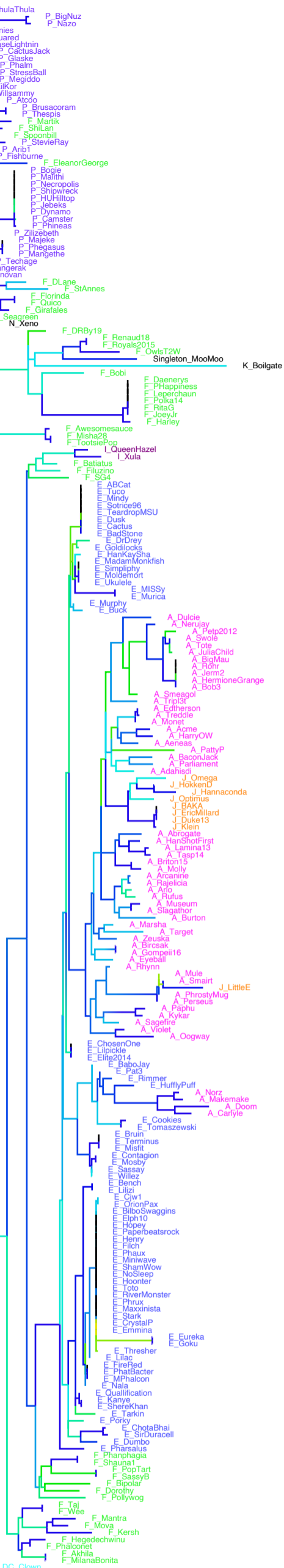

Supplement: Supplementary file 1 [file genes-14-00288-s001.zip › Figure S1B Methylase phylogeny from gappy alignment.treefile.pdf]

### Model Confidence

- Very low (pLDDT < 50)
- Low (70 > pLDDT > 50)
- Confident (90 > pLDDT > 70)
- Very high (pLDDT > 90)

A

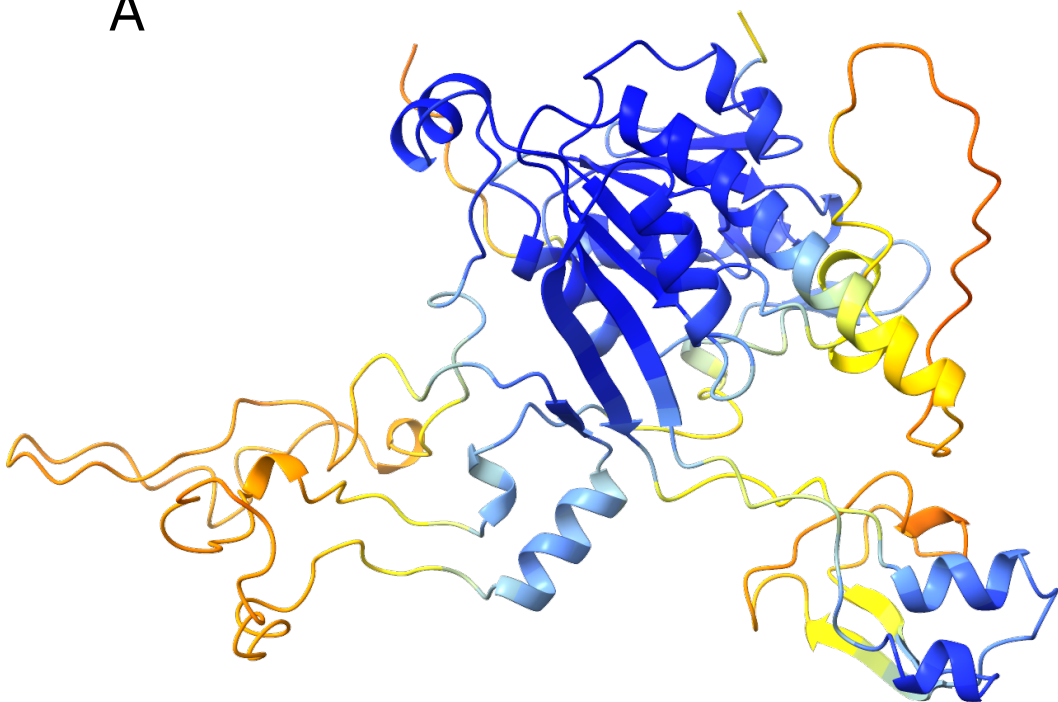

B

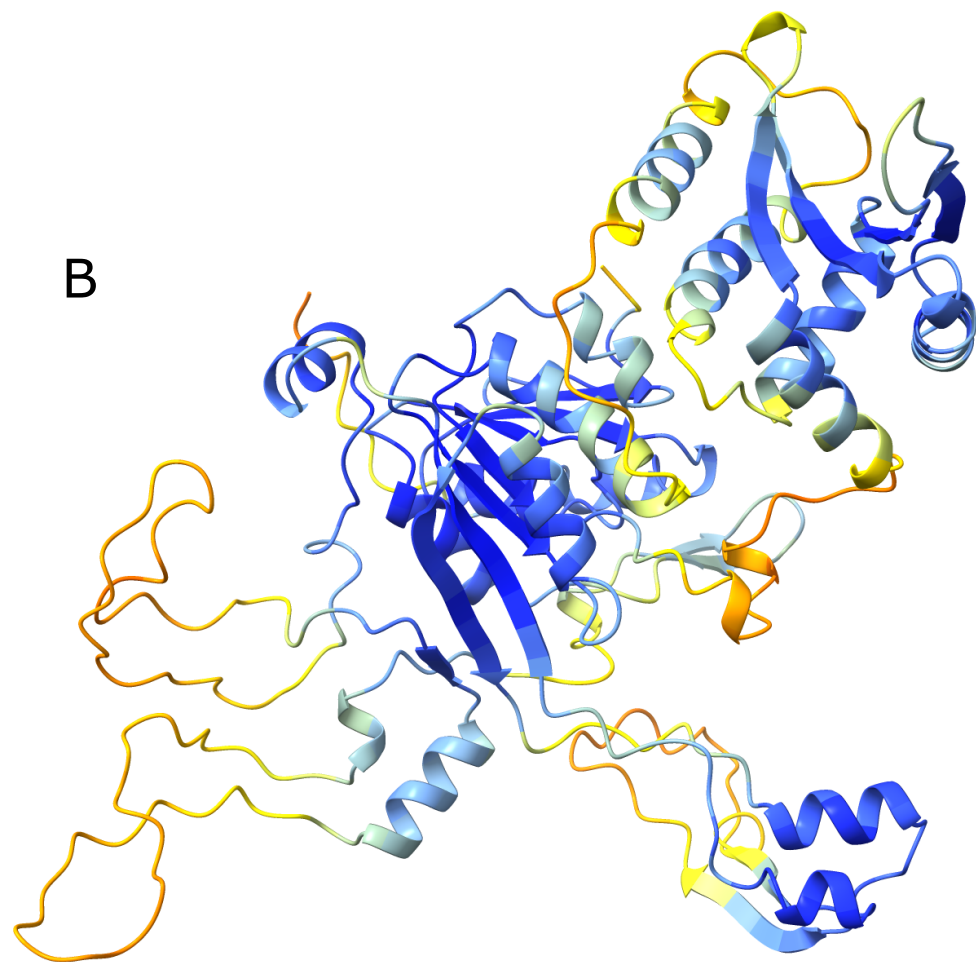

Supplement: Supplementary file 1 [file genes-14-00288-s001.zip › Figure S2 Model Confidence for AlphaFold Predicitons.pdf]
